# Supplementary material for: The crystal structure of vaccinia virus protein E2 and perspectives on the prediction of novel viral protein folds
Source: J Gen Virol. 2022 Jan 12;103(1):001716. doi: 10.1099/jgv.0.001716 (PMC8895614; doi:10.1099/jgv.0.001716)
Supplement: Supplementary material 1 [file jgv-103-1719-s001.pdf]

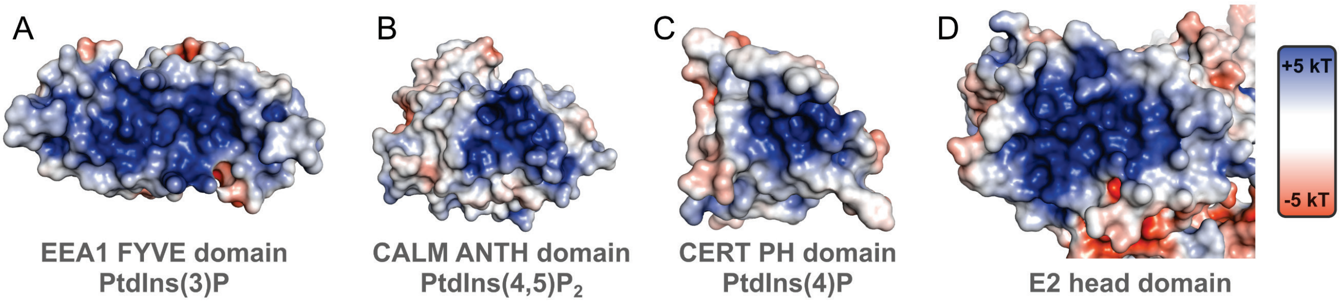

Figure S1. Similarity between VACV E2 head domain basic patch and phosphoinositide-binding cellular domains. Molecular surfaces are coloured by electrostatic potential from red (-5 kT) to blue (+5 kT), as calculated by APBS [1]. (A) The phosphatidylinositol 3-phosphate (PtdIns(3)P)-binding FYVE domain of early endosome antigen (EEA)1, PDB code 1JOC [2]. A dimer of EEA1 FYVE domains, with two PtdIns(3)P binding sites in total, is shown. (B) The phosphatidylinositol 4,5-bisphosphate (PtdIns(4,5)P_2)-binding ANTH domain of CALM, PDB code 3ZYK [3]. (C) The PtdIns(4)P-binding PH domain of CERT, PDB code 4HHV [4]. (D) The putative phosphoinositide binding site on the head domain of VACV E2 (this study).

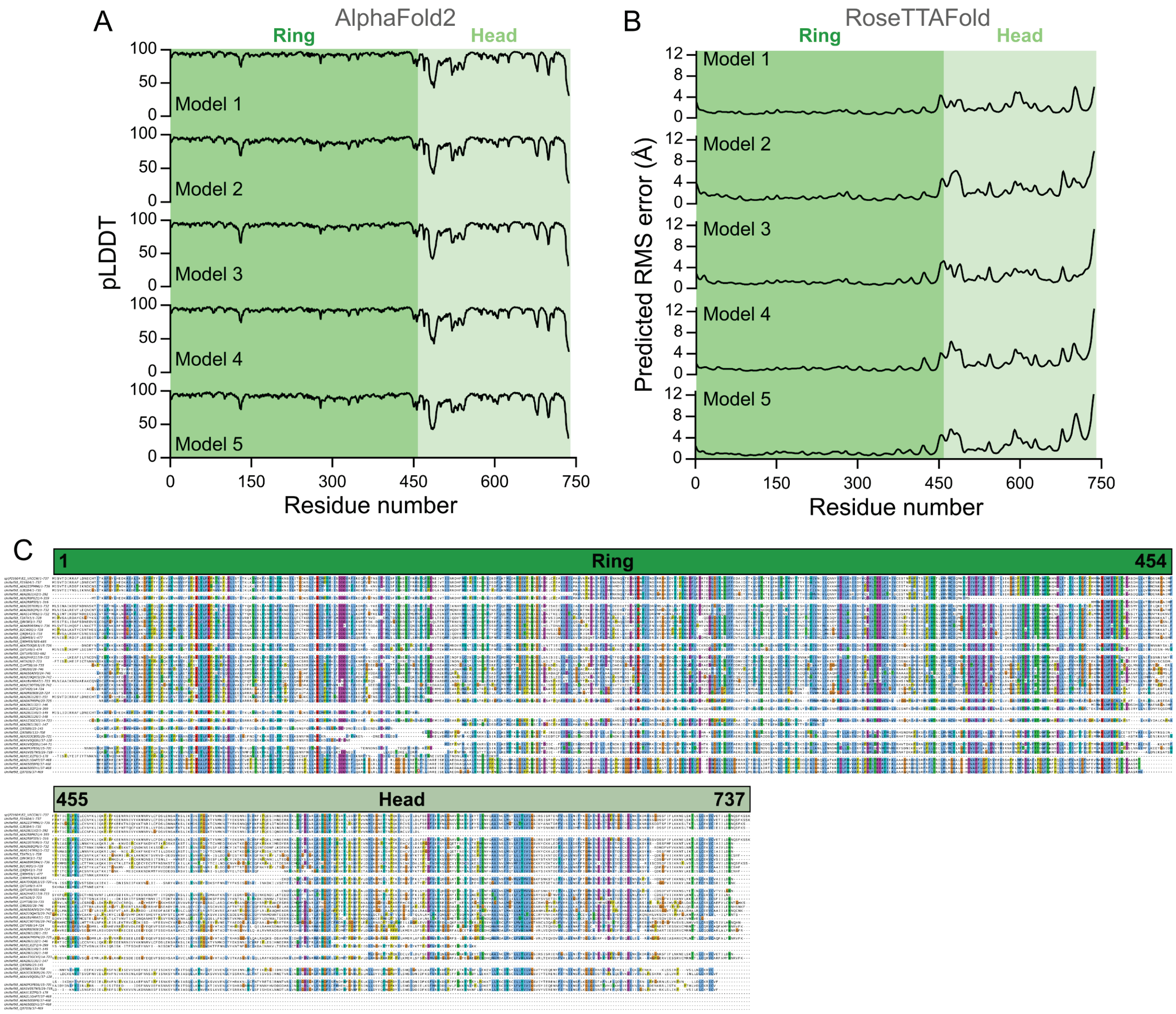

Figure S2. Confidence metrics for predictions of the VACV E2 structure by AlphaFold2 (AF2) and RoseTTAFold (RTF). (A) Plot of per-residue predicted Local Distance Difference Test (pLDDT) for the top five AF2 models. Higher values indicate more confident predictions, with values > 90 being expected to be modelled with high accuracy. (B) Plot of per-residue root mean squared (RMS) error for the top five RTF models. Lower values indicate more confident predictions. (C) Multiple sequence alignment generated by AF2 during prediction of the VACV E2 structure, as described in [5]. Alignment was visualised using Jalview [6] and residues are coloured by residue class ("Clustal" colouring). The displayed sequence alignment was obtained by jackhmmer search of the UniRef90 database, returning 50 homologous sequences. An HHBlits plus HHSearch query of the BFD + Uniclust30 database returned 66 homologous sequences, and a jackhmmer search of the clustered MGnify database returned 5 sequences with a very short stretch of sequence similarity.

Supplementary references

1. **Jurrus E, Engel D, Star K, Monson K, Brandi J, *et al.*** Improvements to the APBS biomolecular solvation software suite. *Protein Sci* 2018;27:112–128.

2. **Dumas JJ, Merithew E, Sudharshan E, Rajamani D, Hayes S, *et al.*** Multivalent endosome targeting by homodimeric EEA1. *Mol Cell* 2001;8:947–958.

3. **Miller SE, Sahlender DA, Graham SC, Honing S, Robinson MS, *et al.*** The molecular basis for the endocytosis of small R-SNAREs by the clathrin adaptor CALM. *Cell* 2011;147:1118–31.

4. **Prashek J, Truong T, Yao X**. Crystal Structure of the Pleckstrin Homology Domain from the Ceramide Transfer Protein: Implications for Conformational Change upon Ligand Binding. *PLoS ONE* 2013;8:e79590.

5. **Jumper J, Evans R, Pritzel A, Green T, Figurnov M, *et al.*** Highly accurate protein structure prediction with AlphaFold. *Nature* 2021;596:583–589.

6. **Waterhouse AM, Procter JB, Martin DMA, Clamp M, Barton GJ**. Jalview Version 2--a multiple sequence alignment editor and analysis workbench. *Bioinformatics* 2009;25:1189–1191.
